# Smallpox

**Published:** 1871-02

**Authors:** 


Smallpox.—A severe epidemic of this disease is now prevailing in London, and is increasing. During the fortnight preceding the 26th of November last, eighty-five deaths from it occurred, and the Registrar-General states that so many weekly deaths from it have not occurred since April, 1867. In the number of the *Lancet* for December 10th, it is stated that the deaths from it during the preceding week had risen to 60, the highest number returned in any week since June, 1863.

It behooves the authorities in this country to take every possible precautionary means of preventing its introduction here, and should it reach us, to prevent its spreading, by general vaccination and re-vaccination. The importance of this last cannot be over-estimated.

